# Multiplexed In-cell Immunoassay for Same-sample Protein Expression Profiling

**DOI:** 10.1038/srep13651

**Published:** 2015-09-02

**Authors:** Jing Shang, Pavel Zrazhevskiy, Nadia Postupna, C. Dirk Keene, Thomas J. Montine, Xiaohu Gao

**Affiliations:** 1 Department of Bioengineering, University of Washington, Seattle, WA 98195, USA; 2 Department of Pathology, University of Washington, Seattle, WA 98195, USA

## Abstract

In-cell immunoassays have become a valuable tool for protein expression analysis complementary to established assay formats. However, comprehensive molecular characterization of individual specimens has proven challenging and impractical due to, in part, a singleplex nature of reporter enzymes and technical complexity of alternative assay formats. Herein, we describe a simple and robust methodology for multiplexed protein expression profiling on the same intact specimen, employing a well-characterized enzyme alkaline phosphatase for accurate quantification of all targets of interest, while overcoming fundamental limitations of enzyme-based techniques by implementing the DNA-programmed release mechanism for segregation of sub-sets of target-bound reporters. In essence, this methodology converts same-sample multi-target labeling into a set of isolated singleplex measurements performed in a parallel self-consistent fashion. For a proof-of-principle, multiplexed detection of three model proteins was demonstrated on cultured HeLa cells, and two clinically-relevant markers of dementia, β-amyloid and PHF-tau, were profiled in formalin-fixed paraffin embedded brain tissue sections, uncovering correlated increase in abundance of both markers in the “Alzheimer’s disease” cohort. Featuring an analytically powerful yet technically simple and robust methodology, multiplexed in-cell immunoassay is expected to enable insightful same-sample protein profiling studies and become broadly adopted in biomedical research and clinical diagnostics.

Quantification of protein expression levels in cell and tissue samples is essential for a variety of biomedical research and clinical applications, such as study of basic cell biology, assessment of drug efficacy and toxicity, association with genetic information, and determination of disease status^1^,^2^,^3^. Expansion of diagnostic biomarker panels and growing complexity of research topics increasingly require a more comprehensive molecular profiling, necessitating development of new technologies for multiplexed quantitative protein analysis^4^,^5^,^6^,^7^. This task has routinely been performed with enzyme-linked immunosorbent assays (ELISA) and western blots, which employ antibodies for specific protein recognition and sensitive enzyme-based reporting mechanism for concentration-dependent signal generation that can be quantified via chemiluminescence, colorimetric, and fluorescence measurements. With appropriate controls and normalization, western blot and ELISA typically offer reliable assessment of protein levels in specimen lysates^8^,^9^. A lysis-free implementation of this technology termed “in-cell ELISA” (also known as in-cell western assay)^10^,^11^ streamlines assay workflow, eliminates potential for protein degradation during lysis, and renders ELISA compatible with hard-to-homogenize specimens, such as archival formalin-fixed paraffin embedded (FFPE) tissues. Therefore, ELISA format provides a robust platform for protein quantification in a wide range of specimens; yet, its capacity for same-sample multiplexed analysis is greatly restricted by the singleplex nature of enzyme-based signal generation.

A number of advanced technologies have been developed to overcome some limitations of enzyme-based assays and tackle the challenges of multiplexed protein expression analysis. For example, microarrays employ spatial segregation of assay “spots” on the same substrate to perform multiple miniaturized singleplexed immunoassays with the same homogenized specimen in parallel^5^,^12^,^13^,^14^. Bead-based assays capture each target protein onto a separate fraction of beads identifiable by a unique size or fluorescent signature for downstream analysis by flow-cytometry or fluorescence imaging in a high-throughput multiplexed manner^15^,^16^,^17^. DNA barcoding methods achieve multiplexing by tagging proteins of interest with a DNA-encoded antibody library and then detecting the unique DNA sequences through polymerase chain reaction (PCR) or fluorescence-based DNA quantification techniques^18^,^19^,^20^,^21^,^22^,^23^. Mass spectrometry offers simultaneous label-free analysis of thousands of target proteins and peptides in homogenized non-crosslinked specimens via detection of protein-specific spectral fingerprints^24^,^25^. Despite great throughput and analytical power of such technologies, however, use of specialized instrumentation, non-trivial preparation of custom assay platforms and reagents, and limited compatibility with different forms of specimens^5^,^26^,^27^,^28^ make substantially more straightforward ELISA and western blot formats still preferable for the majority of current protein analysis applications.

Herein, we describe a simple and robust methodology that combines versatility of ELISA format with a vast encoding capacity of DNA hybridization for multiplexed same-sample protein expression profiling. While retaining many of the components of conventional and in-cell ELISA platforms for broad compatibility with assay reagents and specimen preparations, an inherently singleplex enzyme-based reporting mechanism is rendered multiplexable by introduction of the DNA-programmed release mechanism that enables selective release of target-bound enzyme reporters into solution for subsequent quantification of the released reporter concentration (Fig. 1). Specifically, all surface-bound target proteins (*e.g.*, within formalin-fixed cells) are first simultaneously encoded with unique single-stranded DNA (ssDNA) sequences via recognition by ssDNA-tagged primary antibodies. In contrast to complex and expensive covalent antibody-ssDNA bioconjugation approaches, a non-covalent self-assembly between intact primary antibodies (1′Abs) and ssDNA-linked adaptor protein A^29^ (PrA-ssDNA) is employed to yield a flexible and simple route to on-demand preparation of antibody-ssDNA libraries (1′Ab/PrA-ssDNA). Specimen incubation with a cocktail of complementary ssDNA’-tagged alkaline phosphatase reporters (ssDNA’-AP) simultaneously labels all target proteins with AP via partial DNA hybridization. Finally, addition of a longer “displacement” ssDNA probe breaks complementary Ab-AP links via full hybridization with ssDNA’-AP and triggers “release” of a sub-set of reporters associated with a particular target protein encoded by the “displacement” sequence. Segregation of the released AP fraction allows for quantification of its concentration independent of the reporters remaining on the specimen. Sequential release and segregation of all reporter sub-sets thus allows for analysis of multiple protein targets within the same specimen using an identical reporter enzyme. In essence, this methodology converts same-sample multi-target labeling into a set of isolated singleplex measurements performed in a parallel self-consistent fashion. Importantly, such measurements employ a well-characterized, sensitive, and robust enzyme reporting mechanism and only require instrumentation commonly available in biomedical laboratories.

The performance of the new multiplexed in-cell immunoassay was systematically evaluated on formalin-fixed HeLa cells using three model target proteins—Lamin A, heat shock protein 90 (HSP90), and cytochrome c oxidase 4 (Cox4)—and FFPE brain tissue sections. Two clinically-relevant markers of dementia, β-amyloid and PHF-tau, were profiled in 10 brain tissue sections from an “Alzheimer’s disease” cohort and compared to 9 “Control” specimens.

## Results

### Preparation and characterization of PrA-ssDNA bioconjugates

A library of ssDNA-tagged primary antibodies employed for specific recognition and DNA encoding of molecular targets for the multiplexed in-cell immunoassay was prepared via self-assembly between intact 1′Abs and adaptor PrA-ssDNA bioconjugates. In contrast to prior efforts on molecular engineering of DNA-protein adaptors^22^,^23^,^30^, we used only off-the-shelf components and conventional bioconjugation methodology, making preparation of PrA-ssDNA adaptors and ssDNA-Ab libraries readily accessible to a broad range of laboratories.

**Figure 2:**
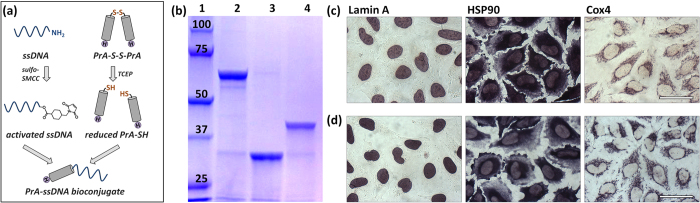
Preparation and characterization of PrA-ssDNA tags. (**a**) Scheme of PrA-ssDNA conjugation procedure. Amine-functionalized ssDNA is activated by sulfo-SMCC, while HIS-tagged cysteine-terminated PrA (supplied in an oxidized PrA-S-S-PrA form) is reduced by TCEP. Activated ssDNA and reduced PrA are reacted to form covalent PrA-ssDNA bioconjugates and purified from the excess ssDNA with HIS SpinTrap column. (**b**) Verification of PrA-ssDNA conjugation and purity with SDS-PAGE: (1) Reference protein ladder (KDa); (2) PrA-S-S-PrA dimer; (3) PrA-SH reduced by TCEP; (4) PrA-ssDNA bioconjugate. (**c,d**) Functionality of PrA-ssDNA tags was assessed by staining of model targets (Lamin A, HSP90, and Cox4) in fixed HeLa cells with pre-assembled 1′Ab/PrA-ssDNA probes followed by complementary ssDNA′-AP (**c**) in comparison to conventional 2-step immunostaining with unmodified 1′Ab followed by 2′Ab-AP probes (**d**). Scale bar, 50 μm.

Specifically, HIS-tagged and cysteine-terminated recombinant PrA was used as a universal adaptor for DNA-tagging of a range of 1′Abs via non-covalent binding to the Fc region of an IgG molecule. C-terminal HIS tag was employed for straightforward probe purification, whereas a single sulfhydryl moiety at the N-terminus was used for 1:1 covalent conjugation with ssDNA sequences. PrA-ssDNA bioconjugation was achieved using common amine-sulfhydryl cross-linking chemistry (Fig. 2a). Amine-functionalized ssDNA was activated by a crosslinker sulfo-SMCC and then reacted with reduced PrA (supplied as a dimer in its oxidized form, PrA-S-S-PrA), forming a stoichiometrically defined linear PrA-ssDNA bioconjugate. The efficiency of the bioconjugation procedure was evaluated with sodium dodecyl sulfate-polyacrylamide gel electrophoresis (SDS-PAGE), which confirmed formation of PrA-SH upon PrA-S-S-PrA dimer reduction by TCEP and showed an increase in molecular weight of the PrA-ssDNA bioconjugate in comparison to unconjugated PrA-SH (Fig. 2b). Importantly, reaction product formed a single sharp band and contained almost no free PrA-SH, featuring the conjugation yield of over 95% at 1:1 PrA-to-ssDNA stoichiometry. High PrA-ssDNA reaction yield at a relatively mild excess of ssDNA (9:1 ssDNA-to-PrA) not only preserved reagents, but also facilitated straightforward 1-step purification with HIS affinity columns, since nearly all of PrA molecules carried ssDNA, whereas lower reaction yield would necessitate additional non-trivial procedure for removal of unconjugated PrA. Having prepared pure PrA-ssDNA adaptors with three distinct DNA sequences, a library of DNA-tagged 1′Abs could be readily assembled by simple mixing of individual unmodified antibodies with respective adaptors at about 10x adaptor excess to ensure complete tagging of all IgG molecules.

### Assessment of target protein labeling, visualization, and quantitative analysis

Capacity of self-assembled 1′Ab/PrA-ssDNA probes for specific binding to intended molecular targets, target protein labeling with ssDNA′-AP reporters via DNA hybridization, and subsequent AP release via DNA displacement was evaluated on formalin-fixed and detergent-permeabilized HeLa cells. While the multiplexed in-cell immunoassay was developed primarily for high-throughput solution-based quantitative analysis of multiple target proteins, use of in-cell labeling methodology and a versatile enzyme-based reporting mechanism also rendered this technology well-suited for immunohistochemistry (IHC)-like singleplex specimen staining with chromogenic substrates. As a result, performance of the assay could be readily assessed in a qualitative manner via brightfield microscopy without requiring substantial modifications to the assay components or methodology.

For singleplex chromogenic cell staining, three model target proteins with distinct intracellular localizations were separately labeled via either 1′Ab/PrA-ssDNA probes followed by ssDNA′-AP (Fig. 2c) or conventional 2-step IHC using unmodified 1′Ab followed by 2′Ab-AP bioconjugates (Fig. 2d). The staining patterns produced by both methods were identical, and all targets demonstrated the expected intracellular localization and lack of non-specific probe binding elsewhere throughout the specimen. Specifically, labeling of Lamin A produced characteristic nuclear membrane staining^31^, highly abundant HSP90 was localized to the cytosol^32^, and Cox4 appeared to be concentrated on the mitochondrial membrane^33^.

For assessment of reporter displacement from the specimen for subsequent solution-based quantitative analysis of target protein abundance, HSP90 was labeled with respective 1′Ab/PrA-ssDNA and ssDNA′-AP probes and then treated with complementary displacement ssDNA probe for 1, 5, 10, 20, and 30 min (Supplementary Figure 1). Concentration of AP in released and specimen-bound fractions was measured using soluble chemiluminescent (CL) substrate on a plate reader. Notably, reporter was readily released into solution by complementary displacement ssDNA probe, with over 90% of AP released within 10 min of incubation, whereas over 99**%** of AP remained bound to the specimen upon incubation with TBS alone. Therefore, assessment of the assay components revealed high specificity of target protein labeling and quick selective release of reporters for solution-based quantitative analysis, supporting the capacity of the multiplexed in-cell immunoassay for accurate profiling of multiple biomarkers in a timely manner.

### Evaluation of Ab/PrA-ssDNA probe stability and cross-reactivity

Specificity and sensitivity of cell staining comparable to that of conventional IHC confirmed the utility of self-assembled Ab/PrA-ssDNA probes and ssDNA′-AP reporters for selective target protein labeling and detection. However, we acknowledge that the universal PrA binding to a range of IgG molecules and the non-covalent nature of PrA/IgG bond carry a risk of Ab/PrA-ssDNA probe dissociation and cross-reactivity between different probes during target labeling. While being only a minor issue for single-target assays, probe cross-reactivity might result in substantial mislabeling of targets in a multiplexed format and yield an incorrect molecular expression profile. Therefore, we carefully investigated the stability of Ab/PrA-ssDNA probes and quantified the extent of probe cross-talk in a multiplexed staining cocktail.

To highlight and quantify potential cross-reactivity between different probes, we devised a modified multiplexed cell labeling procedure, where labeling cocktail contained one pre-assembled 1′Ab/PrA-ssDNA probe mixed with equivalent concentrations of two competing free PrA-ssDNA adaptors. Probes and adaptors were then hybridized with complementary ssDNA′-AP reporters. Specifically, cocktail compositions were: (i) anti-Lamin A Ab/PrA-X1 probe combined with PrA-Y1 and PrA-Z1; (ii) anti-HSP90 Ab/PrA-Y1 with PrA-X1 and PrA-Z1; and (iii) anti-Cox4 Ab/PrA-Z1 with PrA-X1 and PrA-Y1. In such a setting, only pre-assembled 1′Ab/PrA-ssDNA probes are expected to link ssDNA′-AP reporters to the specimen, producing characteristic staining and releasing AP into solution upon DNA displacement, whereas any staining and AP release from competing PrA-ssDNA adaptors would unambiguously indicate probe cross-reactivity (*i.e.*, binding of competing PrA-ssDNA adaptors to 1′Ab during target protein labeling).

**Figure 3:**
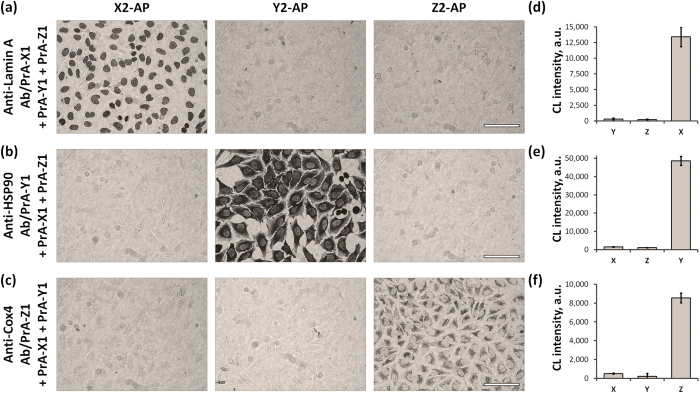
Assessment of cross-reactivity between Ab/PrA-ssDNA probes. Potential probe cross-reactivity was studied qualitatively with optical microscopy (**a–c**) and quantitatively in a multiplexed in-cell immunoassay format (**d–f**). Fixed HeLa cells were incubated with a mixture of one type of Ab/PrA-ssDNA probe and two competing free PrA-ssDNA conjugates: anti-Lamin A Ab/PrA-X1 probe was combined with PrA-Y1 and PrA-Z1 (**a,d**); anti-HSP90 Ab/PrA-Y1 with PrA-X1 and PrA-Z1 (**b,e**); anti-Cox4 Ab/PrA-Z1 with PrA-X1 and PrA-Y1 (**c,f**). For microscopy, single-target staining was performed on separate cell specimens by labeling with either X2-AP, Y2-AP, or Z2-AP probes and reacting with a precipitating chromogenic AP substrate (**a–c**). Images were converted to grayscale for clarity. Scale bar, 100 μm. For multiplexed quantitative analysis, cells were first labeled with all three reporters (a cocktail of X2-AP, Y2-AP, and Z2-AP) and then sequentially treated by displacement probes (X3, Y3, and Z3) to isolate AP fractions corresponding to each DNA sequence. AP concentration in isolated fractions was measured on a plate reader using chemiluminescent (CL) AP substrate (**d–f**). Average values (triplicate experiments normalized by cell number) with standard deviation are shown.

First, cross-reactivity was evaluated qualitatively with brightfield microscopy of chromogenic cell stains (Fig. 3a–c). Cells were treated with an experimental probe cocktail followed by hybridization with only one of the ssDNA′-AP reporters, thus highlighting localization of one of the three PrA-ssDNA adaptors. As expected, even in the presence of competing PrA-ssDNA adaptors, pre-assembled 1′Ab/PrA-ssDNA probes produced correct staining patterns characteristic for corresponding molecular targets: Lamin A (Fig. 3a), HSP90 (Fig. 3b), and Cox4 (Fig. 3c). More importantly, however, is that competing PrA-ssDNA adaptors failed to bind to antibodies already incorporated within Ab/PrA-ssDNA complexes, demonstrating lack of antibody-mediated target protein labeling and, thus, absence of probe cross-reactivity.

Quantitative analysis of cross-reactivity was performed by cell labeling with an experimental probe cocktail, simultaneous hybridization with all ssDNA′-AP probes, and sequential release of each AP reporter with respective displacement probes (Fig. 3d–f). Consistent with qualitative evaluation of cell staining, measurement of the released AP concentrations revealed only trace amounts of reporters in fractions corresponding to competing PrA-ssDNA adaptors, with majority of AP being released from pre-assembled 1′Ab/PrA-ssDNA probes: over 96% for Lamin A (Fig. 3d), over 95% for HSP90 (Fig. 3e), and over 92% for Cox4 (Fig. 3f). At the same time, all target proteins could be reliably quantified, producing signal intensities well within the CL assay linear range (Supplementary Figure 2). Therefore, qualitative evaluation and quantitative analysis of the probe cross-reactivity confirmed sufficient stability of pre-assembled Ab/PrA-ssDNA probes and highlighted the lack of cross-reactivity even in the presence of a large excess of competing PrA-ssDNA adaptors, supporting the utility of this assay for target protein detection and quantification in a multiplexed format.

### Multiplexed quantification of three model biomarkers in HeLa cells

Having confirmed target labeling specificity and lack of potential cross-reactivity between different probes, we proceeded to assay validation in formalin-fixed detergent-permeabilized HeLa cells. Three model biomarkers—Lamin A, HSP90, and Cox4—were simultaneously labeled by a cocktail of 1′Ab/PrA-ssDNA probes followed by a cocktail of ssDNA′-AP reporters. Lamin A was encoded by sequence “X”, HSP90 by “Y”, and Cox4 by “Z”. Then, target-bound reporters were released into solution one-by-one via DNA bond displacement, AP concentration in released fractions was measured with CL substrate on a plate reader, and signals were normalized to yield the relative biomarker abundance in cells (Table 1). Substantially higher levels of a ubiquitous cytoplasmic target HSP90 in comparison to less abundant organelle-associated proteins Lamin A (nuclear membrane) and Cox4 (mitochondria) was observed. Importantly, same-sample molecular profile obtained with the multiplexed assay format was completely consistent with reference single-target measurements recorded from separate specimens, and the order of reporter displacement did not affect the measurements, as no statistically-significant differences were found between biomarker abundance levels recorded from displacement orders (i) X→ Y→ Z, (ii) Y→ Z→ X, and (iii) Z→ X→ Y as well as single-target assays (one-way ANOVA P > 0.05 for all biomarkers). Thus, the results confirmed that multiplexed target protein encoding and labeling followed by DNA-mediated reporter release could produce accurate relative molecular profiles of formalin-fixed cells.

### Protein expression profiling on FFPE brain tissue sections

**Figure 4:**
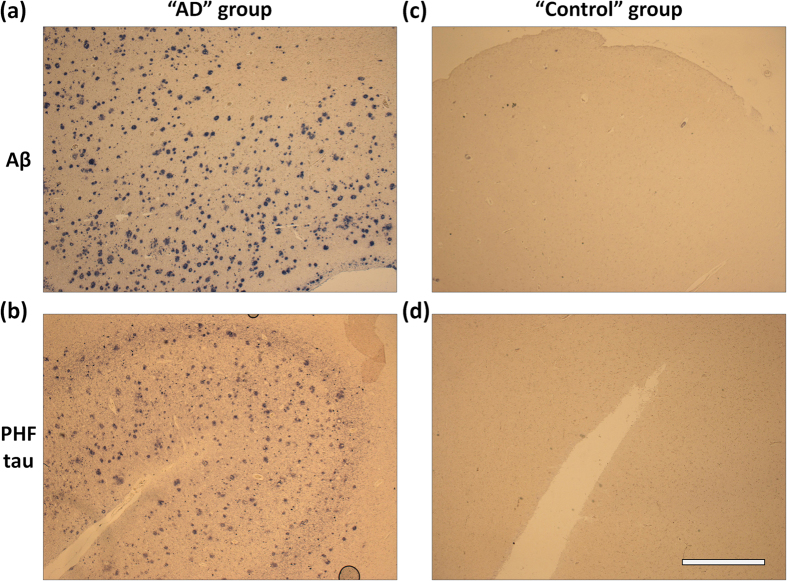
IHC staining of FFPE brain tissue sections with Ab/PrA-ssDNA probes. Representative FFPE brain tissue sections from “Alzheimer’s disease (AD)” (**a,b**) and healthy “Control” (**c,d**) groups were labeled for either Aβ (**a,c**) or PHF-tau (**b,d**) pathologic proteins with corresponding 1′Ab/PrA-ssDNA probes and complementary ssDNA′-AP reporters. Localization of labeled proteins was highlighted by a precipitating chromogenic AP substrate (dark spots) and examined via brightfield microscopy. Scale bar, 1 mm.

Robustly quantitative analysis of multiple biomarkers within FFPE tissue sections has potentially enormous diagnostic and clinical research value, but established methods do not exist. Thus, we also validated the utility of the multiplexed in-cell immunoassay for profiling of clinical tissue specimens. As a model for this study we chose Alzheimer’s disease (AD), a serious neurodegenerative disorder associated with accumulation of β-amyloid (Aβ) and paired helical filament (PHF)-tau^34^,^35^ in the brain. Both pathologic proteins could be reliably stained via 1′Ab/PrA-ssDNA probes and ssDNA′-AP reporters in brain tissue sections from patients with AD forming characteristic patterns^36^,^37^ of amyloid plaques (Fig. 4a and Supplementary Figure 3a) and neurofibrillary tangles (Fig. 4b and Supplementary Figure 3b), but were absent in the cohort of “Control” individuals who were not diagnosed with dementia during life and who did not meet pathologic criteria for AD (Fig. 4c,d and Supplementary Figure 3c,d), giving us confidence in the specificity of target protein labeling and corroborating the utility of our method for accurate biomarker quantification.

**Figure 5:**
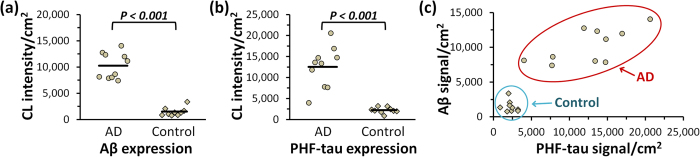
Same-sample quantitative analysis of Aβ and PHF-tau abundance in brain tissue sections. FFPE brain tissue sections from “Alzheimer′s disease (AD)” (n = 10) and healthy “Control” (n = 9) groups were simultaneously labeled for Aβ and PHF-tau pathologic proteins. AP reporters corresponding to each target were sequentially released via DNA displacement and measured with a plate reader. Chemiluminescence signal was normalized by the gray matter area on each tissue section. (**a**) Comparison of Aβ expression in “AD” and “Control” groups. (**b**) Comparison of PHF-tau expression in “AD” and “Control” groups. Average expression level is indicated by a solid line on each scatter plot. (**c**) Pairwise comparison of Aβ and PHF-tau expression in “AD” (circles) and “Control” (diamonds) groups.

Assay validation was performed by simultaneous same-sample labeling and quantification of Aβ and PHF-tau in FFPE brain tissue sections from 19 subjects—10 from the AD cohort and 9 from the control group (Fig. 5). As expected, all “Control” specimens were negative for both pathologic proteins, whereas the AD group yielded significantly elevated levels of Aβ and PHF-tau (two-tailed t-test P < 0.001) consistent with the molecular manifestation of Alzheimer′s disease. Greater variation in protein abundance was also observed among different AD cases, which, given similar disease stage, might indicate involvement of different mechanisms in disease pathogenesis. More importantly, the multiplexed in-cell immunoassay produced quantitative data for both pathologic proteins from the same specimen, enabling direct comparison and correlation of protein expression levels (Fig. 5c).

## Discussion

In-cell immunoassays have become a valuable tool for protein quantification, particularly in cases when the target state might be altered by processing (*e.g.*, enzyme phosphorylation), or the specimen is not amenable to homogenization (*e.g.*, clinical FFPE specimens). Use of reporter enzymes contributes to sensitivity and simplicity of the method, which can be readily implemented by a wide range of biomedical laboratories. The singleplex nature of enzyme-based signal development, however, limits this technique to quantitative analysis of only one protein per specimen, requiring multiple samples and additional signal normalization and comparison across samples for more comprehensive molecular profiling. Data pooled from inevitably heterogeneous samples might introduce undesirable variability and errors. Furthermore, multiple comparable samples of the same specimen are often not available for analysis (*e.g.*, rare cells, biopsies). To overcome this limitation, yet retain the advantages of in-cell immunoassay format, we have developed a multiplexed in-cell immunoassay that employs target protein encoding with unique DNA tags, simultaneous labeling of all target proteins with the same reporter enzyme, and sequential sequence-specific DNA-mediated release of reporters associated with individual target proteins. As a result, a multiplexed analysis can be performed on the same sample using the same reporter enzyme in a robust, self-consistent manner. To demonstrate the concept we have profiled 3 model target proteins in HeLa cells and two pathologic proteins in FFPE brain tissue sections with a single reporter enzyme AP. Given the generally high specificity of primary antibodies and vast encoding capacity of DNA hybridization, the multiplexed in-cell immunoassay platform described here should be readily extended to profiling of tens of proteins within the same sample, while being limited by the antibody cross-reactivity analogously to other multiplexed immunoassay (such as microarrays and bead-based assays)^38^.

Multiplexed in-cell immunoassay relies on accurate encoding of individual molecular targets with unique DNA sequences via immunorecognition by ssDNA-tagged primary antibodies. To date, preparation of DNA-encoded antibody libraries has been explored in detail for a variety of applications, including immuno-PCR^22^, cell isolation^20^, protein detection^23^, and imaging^39^. However, probe preparation remains a prohibitive hurdle to broader adoption of this technology, requiring a complex and expensive covalent antibody-DNA bioconjugation or custom engineering of adaptor molecules. Realizing this limitation, we have devised a probe preparation methodology using only off-the-shelf components. Specifically, sulfhydryl-terminated protein A is covalently conjugated to an amine-terminated ssDNA tag via conventional cross-linking, producing a linear PrA-ssDNA adaptor capable of binding a variety of unmodified IgG antibodies to produce self-assembled Ab/PrA-ssDNA probes. Notably, defined 1:1 stoichiometry and linear structure of PrA-ssDNA bioconjugates preserve IgG-binding domains of PrA required for stable probe assembly^29^, while limiting the number of ssDNA tags that are deposited on each antibody, minimizing potential off-target nuclear binding by ssDNA tags and rendering our methodology compatible with incubation buffers and conditions routinely employed for immunoassays. Proof-of-concept studies on cultured formalin-fixed HeLa cells confirmed sufficient stability and specificity of self-assembled Ab/PrA-ssDNA probes, corroborating the use of this straightforward probe preparation strategy for multiplexed target labeling.

Selective and fast reporter release represents another essential component of the multiplexed in-cell immunoassay. Timely DNA bond displacement is desirable for efficient segregation and analysis of all probe fractions in a multiplexed format, whereas sufficient selectivity of displacement is necessary to avoid undesirable release of multiple probe fractions at one time. In contrast to solution-based sorting^40^, where over 97% displacement yield could be achieved within 1 minute of incubation, we have found that release of surface-bound reporter enzymes is typically incomplete and proceeds at a slower pace, yielding about 80% displacement yield after a 1-minute incubation and taking up to 10 minutes for release of 90% of reporters. Remaining reporters likely stay irreversibly bound to the specimen and do not get released into subsequent fractions, as demonstrated by the lack of release order effect on the recorded molecular profile. At the same time, there was no substantial non-specific displacement by non-complementary ssDNA probes within this timeframe, supporting the use of 10-minute reporter release steps.

Among many possible applications, multiplexed in-cell immunoassay is expected to make a major impact on analysis of archival FFPE tissue specimens for research and diagnostic purposes. Conventional assessment of pathologic proteins or biomarkers in tissue sections typically relies on semi-quantitative and somewhat subjective visual scoring of immunohistochemical (IHC) tissue stains^41^,^42^. This limitation has prompted development of new techniques capable of objective and quantitative protein analysis. One such technique, Histelide^37^,^43^, combines in-cell ELISA and IHC to eliminate potential inaccuracies caused by visual scoring and generates standardized quantitative results. However, similarly to other in-cell immunoassays, Histelide lacks the multiplexing capability for molecular profiling on the same tissue slide. Incorporation of the DNA-mediated encoding and sorting strategy within in-cell ELISA format greatly expands assay capacity for simultaneous quantification of multiple targets, while retaining compatibility with common FFPE specimen processing workflow. In fact, multiplexed protein expression profiling employs, with minimal alteration, all the steps of typical IHC procedures used in clinical research settings, adding only 10 minutes per target protein to the overall assay time. To demonstrate this concept, we have performed simultaneous quantification of clinically-relevant markers of AD, β-amyloid and PHF-tau, in FFPE brain tissue sections, recording accumulation of both pathologic proteins in AD cohort brain sections and near absence of these targets in Control cohort specimens, in agreement with previous studies^37^. Further, we emphasize the benefit of multiplexed assay format for direct comparison of biomarker profiles within the same specimen, which might uncover surprising relationships between different biomarkers that may not be evident in single-target studies. Given the versatility of probe preparation and vast encoding capacity of DNA tags, we believe this methodology can be readily expanded to a larger set of biomarkers and applied to other specimens.

In summary, the multiplexed in-cell immunoassay described here uniquely enables same-sample protein expression profiling in a highly accessible and robust manner by implementing the DNA-programmed reporter release mechanism within an ELISA format. Essentially, our methodology converts same-sample multi-target labeling into a set of isolated singleplex measurements performed in a parallel self-consistent fashion, taking advantage of the beneficial features of ELISA, but greatly expanding its analytical power. Methodology simplicity and compatibility with common specimen processing procedures are added advantages that give this multiplexed in-cell immunoassay instant utility for numerous forms of same-sample protein expression profiling research and diagnostic applications.

## Methods

### Materials

Protein A with C-terminal cysteine and N-terminal HIS-tag (PrA-SH) was purchased from Abcam. Monoclonal rabbit antibody against Cox4 (Cell signaling Technology), polyclonal rabbit antibody against Lamin A (Sigma Aldrich), and monoclonal rabbit antibody against HSP90 (Epitomics) were used for labeling of cultured cells. Polyclonal rabbit antibodies against β-amyloid (Aβ, Abcam) and paired helical filament–tau (PHF-tau, LifeSpan BioSciences) were used for immunoassays on brain tissue sections. Reference two-step immunolabeling was done with secondary goat anti-rabbit IgG-alkaline phosphatase (2′Ab-AP, Life Technologies). DNA probes were designed as described previously^40^ and synthesized by Integrated DNA Technologies. Encoding probes (X1, Y1, Z1) were functionalized with a primary amine group at the 5′ end for covalent conjugation to PrA-SH. Reporter probes (X2, Y2, Z2) were functionalized with a 5′ biotin tag for self-assembly with streptavidin-alkaline phosphatase (SAv-AP, Life Technologies). Encoding and reporter probe pairs have 16-bp complementarity, whereas displacement probes (X3, Y3, Z3) have longer 22-bp complementarity to reporter probes. All DNA probes were purified with HPLC, reconstituted in DNase-free water (Thermo Scientific) at 100 μM, and stored at −20 °C. Sequences of DNA probes are:

X1: 5′-AAAAAAAAAA**ACGTATGGCAAGTCTA**-3′

X2: 5′-AAAAAAAAAATGTGAA**TAGACTTGCCATACGT**-3′

X3: 5′-**ACGTATGGCAAGTCTA**TTCACA-3′

Y1: 5′-AAAAAAAAAA**CCTGGTCTCAAGAATT**-3′

Y2: 5′-AAAAAAAAAATACCGT**AATTCTTGAGACCAGG**-3′

Y3: 5′-**CCTGGTCTCAAGAATT**ACGGTA-3′

Z1: 5′-AAAAAAAAAA**AGATGACGCTAGGGAA**-3′

Z2: 5′-AAAAAAAAAAGCATTG**TTCCCTAGCGTCATCT**-3′

Z3: 5′-**AGATGACGCTAGGGAA**CAATGC-3′

### Probe preparation

PrA-ssDNA conjugates were prepared via maleimide-mediated crosslinking between reduced PrA-SH and activated ssDNA^30^. First, encoding ssDNA sequences were activated by Sulfo-SMCC (Thermo Scientific): 100 μl of ssDNA (100 μM) in phosphate buffered saline (PBS, pH 7.4) was mixed with 13.6 μl Sulfo-SMCC (20 mg/ml) in dimethyl sulfoxide and incubated for 1 h at room temperature. At the same time, PrA-SH was reduced by mixing 1 mg/ml PrA with (tris(2-carboxyethyl)phosphine) (TCEP, 2 mM) and incubating for 45 min at 37 °C. Activated ssDNA and reduced PrA-SH were purified by Zeba desalting spin columns (Thermo Scientific), mixed together at ssDNA-to-PrA molar ratio of 9:1, and reacted for 3 h at room temperature. PrA-ssDNA conjugates were purified using His SpinTrap columns (GE Healthcare). Successful conjugation was verified by SDS-PAGE using 10% Precise Protein Gel and Tris-HEPES running buffer (Thermo Scientific). Antibody/PrA-ssDNA probes were prepared prior to specimen labeling by simple mixing of primary antibodies (1′Ab) with PrA-ssDNA conjugates at 1:10 Ab-to-PrA molar ratio and incubating for 1 h at room temperature. Detection ssDNA′-AP probes were prepared prior to specimen labeling by first incubating biotinylated ssDNA reporter probes with SAv-AP at approximately 5:1 ssDNA-to-SAv molar ratio for 30 min and then removing unbound ssDNA with SAv-coated magnetic beads (Life Technologies) for 30 min. All self-assembly reactions were performed at highest available reagent concentrations, and probes were diluted to a working concentration immediately prior to specimen labeling.

### Cell culture and processing

Human cervical cancer cell line HeLa (ATCC) was used as a model for evaluation of the multiplexed in-cell immunoassay performance. Cells were grown in glass-bottom 24-well plates (Greiner Bio-One) in a humidified atmosphere at 37 °C with 5% CO_2_ to a density of 80% using MEM culture medium with L-glutamine (Invitrogen) supplemented with 10% fetal bovine serum (PAA Laboratories) and antibiotics (60 mg/ml streptomycin and 60 U/ml penicillin). Prior to labeling, cells were rinsed with tris-buffered saline (TBS, pH 7.4), fixed with 4% formaldehyde in TBS for 20 min, permeabilized with 2% DTAC (dodecyltrimethylammonium chloride, Sigma-Aldrich) in TBS for 20 min followed by 0.25% TritonX-100 (Thermo Scientific) in TBS for 5 min, and washed with TBS. Fixed cells were stored in TBS with 0.03% sodium azide at 4 °C.

### Evaluation of the Antibody/PrA-ssDNA probe binding specificity

Binding specificity was assessed by comparing staining patterns of Lamin A, HSP90, and Cox4 in HeLa cells obtained via (i) DNA-mediated labeling and (ii) reference 2-step immunolabeling. Cells were blocked with a blocking buffer (2% BSA (Bovine serum albumin, Sigma Aldrich), 0.5% dextran sulfate (Sigma Aldrich), 0.05% Tween-20 (Thermo Scientific), and 0.5 mg/ml shredded salmon sperm DNA (Life Technologies) in TBS) for 30 min and incubated with approximately 4 nM unmodified 1′Ab or pre-assembled 1′Ab/PrA-ssDNA probes in blocking buffer for 1 h at room temperature. Cells were then washed with TBS and labeled for 1 h with 2′ Ab-AP conjugates or ~9 nM pre-assembled ssDNA′-AP probes in labeling buffer (2% BSA, 0.5 mg/ml shredded salmon sperm DNA, TBS), respectively. Following washing, cells were processed for microscopy.

### Evaluation of the Antibody/PrA-ssDNA probe stability and cross-reactivity

Stability of pre-assembled Ab/PrA-ssDNA probes and lack of cross-talk in a multiplexed labeling format was confirmed by performing single-target labeling with one pre-assembled 1′Ab/PrA-ssDNA probe in the presence of the other two free PrA-ssDNA conjugates of equivalent concentration that served as competitors for 1′Ab binding. Cells were incubated with the following cocktails in blocking buffer for 1 h: (i) anti-Lamin A Ab/PrA-X1 with free PrA-Y1 and PrA-Z1; (ii) anti-HSP90 Ab/PrA-Y1 with free PrA-X1 and PrA-Z1; (iii) anti-Cox4 Ab/PrA-Z1 with free PrA-X1 and PrA-Y1; and (iv) free PrA-X1, PrA-Y1, and PrA-Z1 as a control. Subsequently, cells were labeled by a cocktail of X2, Y2, and Z2 pre-assembled ssDNA′-AP probes in labeling buffer for 1 h. For qualitative evaluation of labeling specificity and cross-talk, cells were further processed for microscopy. For quantitative analysis (in a separate experiment), ssDNA′-AP probes were sequentially released from cells into solution by 10-min incubations with displacement DNA probes and further processed for measurement with a plate reader. To rule out artifacts introduced by the order of displacement, ssDNA′-AP probes bound to competitor PrA-ssDNA conjugates were released first, followed by AP release from Ab/PrA-ssDNA probes. To account for differences in cell counts between triplicates, measurements were normalized by total cell numbers using Janus Green whole cell stain (Thermo Scientific).

### Multiplexed quantification of molecular targets in fixed cells

Analogously to the single-target labeling procedure described above, multiplexed quantitative analysis on the same sample was achieved by first incubating cells with a cocktail of three pre-assembled 1′Ab/PrA-ssDNA probes (*i.e.*, anti-Lamin A Ab/PrA-X1, anti-HSP90 Ab/PrA-Y1, and anti-Cox4 Ab/PrA-Z1) for 1 h, followed by labeling with a cocktail of three pre-assembled ssDNA′-AP probes for 1 h and thorough washing with TBS. Individual ssDNA′-AP probes were then released into solution via sequence-specific 10-min displacement with complementary probes X3, Y3, and Z3 and processed for measurement with a plate reader. To validate the accuracy of relative protein levels obtained from the same sample through the DNA displacement method, each of the model targets (Lamin A, HSP90, and Cox4) was individually labeled with respective 1′Ab/PrA-ssDNA and ssDNA′-AP probes in separate cell samples and processed for measurement with a plate reader without displacement, as in a typical in-cell ELISA. To account for differences in cell counts between specimens in this case, measurements were normalized by total cell numbers using Janus Green whole cell stain (Thermo Scientific).

### Quantification of proteins in FFPE tissue specimens

This study was performed in accordance with the University of Washington Institutional Review Board (UW IRB) guidelines and oversight. All cases were from the UW Neuropathology Core; all neuropathologic evaluations of autopsy material were performed using consensus protocols and methods. Superior and middle temporal gyrus blocks were dissected at autopsy, embedded in paraffin, and sectioned onto glass slides. All subjects were divided into 2 groups based on a clinical and neuropathologic diagnosis of AD. The AD group included 10 subjects with neuropathologic diagnosis of severe AD neuropathologic change according to current criteria^44^,^45^. The control group included 9 subjects with none or low AD neuropathologic change. All 19 selected cases had two or fewer cerebral microinfarcts and no Lewy body disease. The post-mortem interval to fixation was less than eight hours for all cases. Tissue slides were deparaffinized and rehydrated following previously described protocol^37^. Briefly, slides were washed in 4 changes of xylene, 2 changes of xylene/isopropanol 1:1 mixture, and hydrated by graded isopropanol washes in series (100%, 100%, 96%, 70%, 50%) and TBST (10 mM Tris-HCl pH 7.8, 100 mM NaCl, 0.05% Tween-20), followed by antigen retrieval in 88% formic acid for 7 min, further washing with TBST, and blocking in 2% BSA with 0.5% casein in TBST at 4 °C overnight. The pre-blocked slides were incubated in a cocktail solution containing pre-assembled anti-PHF-tau Ab/PrA-X1 and anti-Aβ Ab/PrA-Y1 in 2% BSA/0.5% dextran sulfate/0.05% Tween-20/0.5 mg/ml shredded salmon sperm DNA/TBS for 2 h at room temperature, washed with TBST, and labeled with a cocktail of X2-AP and Y2-AP in 2% BSA/0.5 mg/ml shredded salmon sperm DNA/TBS for 1 h. Following washing with TBST, individual probes were sequentially released by addition of X3 and Y3 sequences for measurement with a plate reader. To account for differences in tissue section sizes, data were normalized by the gray matter area on each slide. Optionally, further single-target staining was performed by again incubating the specimen with one type of ssDNA′-AP probes (*i.e.*, X2 for PHF-tau staining or Y2 for Aβ staining) for 1 h and processing for microscopy.

### Microscopy

Qualitative evaluation of cell labeling was done by bright-field microscopy on an inverted Olympus IX71 microscope equipped with a true-color QColor5 digital camera (Olympus). Low-magnification images were obtained with ×4 dry objective (NA 0.13, Olympus) and high-magnification with ×40 oil-immersion objective (NA 1.30, Olympus) and ×100 oil-immersion objective (NA 1.40, Olympus). AP-mediated deposition of a precipitating substrate 5-Bromo-4-Chloro-3-Indolyl Phosphate/Nitroblue Tetrazolium (BCIP/NBT, Life Technologies) was used for label visualization.

### Chemiluminescence signal measurement and statistical analysis

Quantitative analysis of target abundance was done by measuring AP-mediated chemiluminescence (CL) of CSPD substrate with Emerald-II enhancer (Life Technologies) using Infinite M200 plate reader (Tecan). A background signal arising, in part, from spontaneous CL and non-specific PrA-ssDNA specimen binding was subtracted from all measurements. Relative protein abundance was calculated by normalizing absolute CL values obtained for each protein to a sum of CL values for all three target proteins. Cell labeling experiments were performed in triplicates, and data were expressed as an average (n = 3) with standard deviation. Statistical analysis of the HeLa cell protein profiling data was performed with one-way ANOVA (sample size n = 3). Statistical analysis of tissue specimen data was done using unpaired two-tailed t-test (sample size n = 10 for “AD” group and n = 9 for “Control” group).

## Additional Information

**How to cite this article**: Shang, J. *et al.* Multiplexed In-cell Immunoassay for Same-sample Protein Expression Profiling. *Sci. Rep.* **5**, 13651; doi: 10.1038/srep13651 (2015).

## Supplementary Material

Supplementary Information

## Figures and Tables

**Figure 1:**
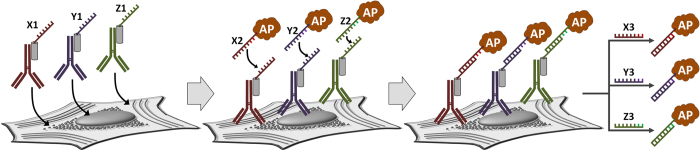
Schematic illustration of the multiplexed in-cell immunoassay. The specimen is first incubated with a library of self-assembled 1′Ab/PrA-ssDNA probes and then with a cocktail of complementary ssDNA′-functionalized reporters (*e.g.*, an enzyme alkaline phosphatase, AP). Target labeling occurs through (i) antibody-antigen binding and (ii) ssDNA-ssDNA′ hybridization. As a result, all targets of interest are simultaneously labeled by the same reporter, but each target is linked to a reporter via a displaceable DNA bridge with a unique sequence (*e.g.*, X1-X2, Y1-Y2, or Z1-Z2). This enables the release of subsets of target-bound reporters into solution via sequence-specific DNA bond displacement with a longer complementary ssDNA probe (X3, Y3, or Z3, respectively), yielding unbound reporter concentration equivalent to the corresponding target abundance in the specimen. Multiplexed same-sample protein expression profiling is achieved by quick sequential release and segregation of all target-bound reporters one-by-one (*e.g.*, into separate wells of a 96-well plate for high-throughput quantitative analysis with a plate reader).

**Table 1:** Relative abundance of proteins in HeLa cells detected by the multiplexed immunoassay via DNA-mediated reporter release in comparison to single-target on-surface analysis.

Method	Reporter release sequence	**Protein abundance (%)**		
		**Lamin A (X)**	**HSP90 (Y)**	**Cox4 (Z)**
Multiplexed immunoassay via DNA-mediated reporter release	X - > Y - > Z	17.7 ± 1.9	69.7 ± 2.7	12.6 ± 0.9
	Y - > Z - > X	21.1 ± 1.5	65.8 ± 1.4	13.1 ± 1.2
	Z - > X - > Y	19.1 ± 5.9	68.9 ± 5.3	12.0 ± 0.6
Single-target immunoassay on 3 separate samples*	N/A	19.7 ± 1.5	68.1 ± 1.8	12.2 ± 0.3

Average results from triplicate experiments ± s.d. are shown.

One-way ANOVA (among the three multiplexed assays and a corresponding single-target assay for each protein) P-value = 0.66 for Lamin A (X) group, 0.51 for HSP90 (Y), and 0.42 for Cox4 (Z).

^*^Signals were normalized by total cell numbers from each sample.
