# Supplementary material for: Multiplexed In-cell Immunoassay for Same-sample Protein Expression Profiling
Source: Sci Rep. 2015 Sep 2;5:13651. doi: 10.1038/srep13651 (PMC4556981; doi:10.1038/srep13651)
Supplement: Supplementary Information [file srep13651-s1.pdf]

SUPPLEMENTARY INFORMATION

Multiplexed In-cell Immunoassay for Same-sample Protein Expression Profiling

Jing Shang[1,†], Pavel Zrazhevskiy[1,†], Nadia Postupna[2], C. Dirk Keene[2], Thomas J. Montine[2,*], and Xiaohu Gao[1,*]

[1] Department of Bioengineering, University of Washington, Seattle, WA 98195, USA.
[2] Department of Pathology, University of Washington, Seattle, WA 98195, USA.
† These authors contributed equally.
***** Correspondence should be addressed to T.J.M. (tmontine@uw.edu) and X.H.G. (xgao@uw.edu).

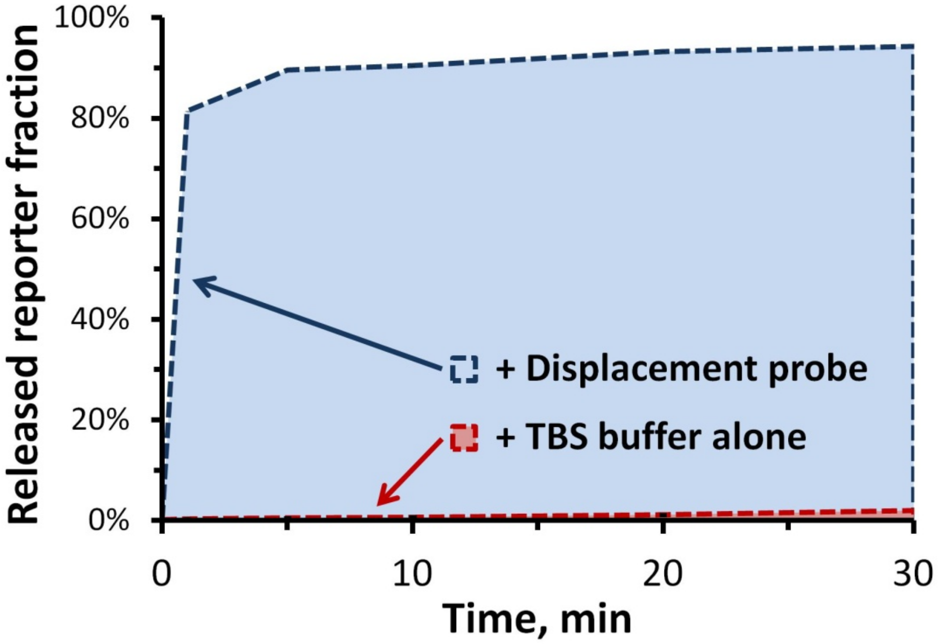

Supplementary Figure 1. Assessment of the AP release kinetics via DNA-mediated bond displacement. HSP90 in fixed HeLa cells was first labeled with anti-HSP90 Ab/PrA-Y1 and Y2-AP probes. Then, AP was released from the specimen into solution by addition of Y3 displacement probe for 1, 5, 10, 20, and 30 min (in separate wells). Non-specific probe release was assessed by incubating cells with TBS alone. CL signal from the released AP as well as AP remaining bound to the specimen was measured. To account for the lower enzymatic activity of a surface-bound AP, CL signal from the specimen-bound AP fraction was multiplied by a factor of 1.56 (determined in a separate experiment comparing CL signal generated by the same concentration of AP bound to the surface vs. dispersed in solution). Fraction released was calculated as a ratio of the signal from AP in solution to the total signal from both AP in solution and on the specimen.

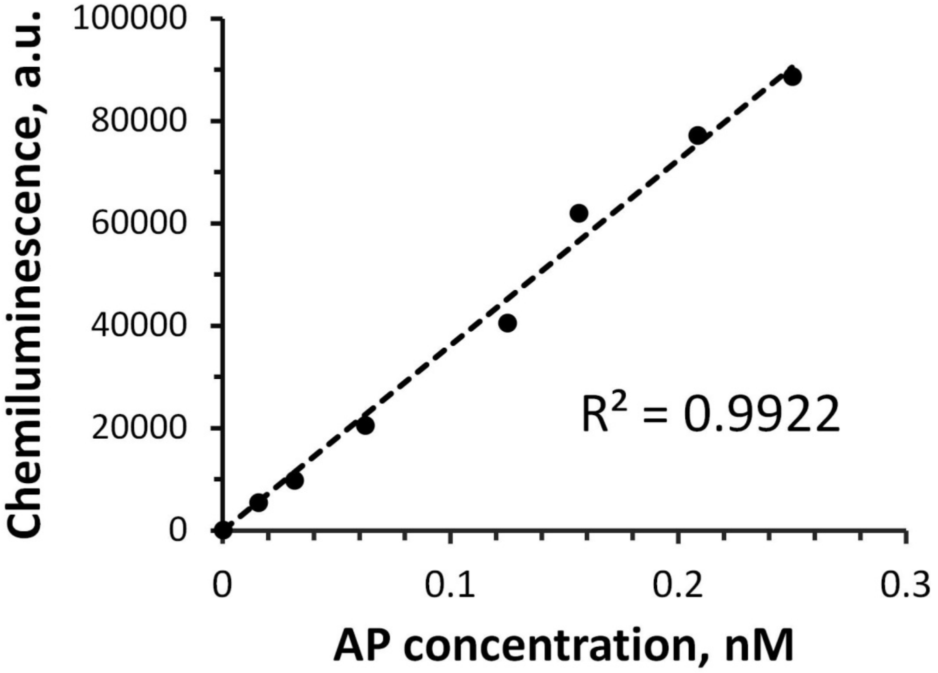

Supplementary Figure 2. Validation of the linear working range of chemiluminescence assay for quantification of AP concentration. AP reporter was serially diluted in TBS from 0.25 nM down to 0.015 nM concentration, incubated with a soluble chemiluminescent substrate, and measured with a plate reader. Linear relationship between the AP concentration and the chemiluminescence signal (linear fit R^2=0.99) was observed for a range of concentrations and CL intensities consistent with the same-sample protein profiling studies.

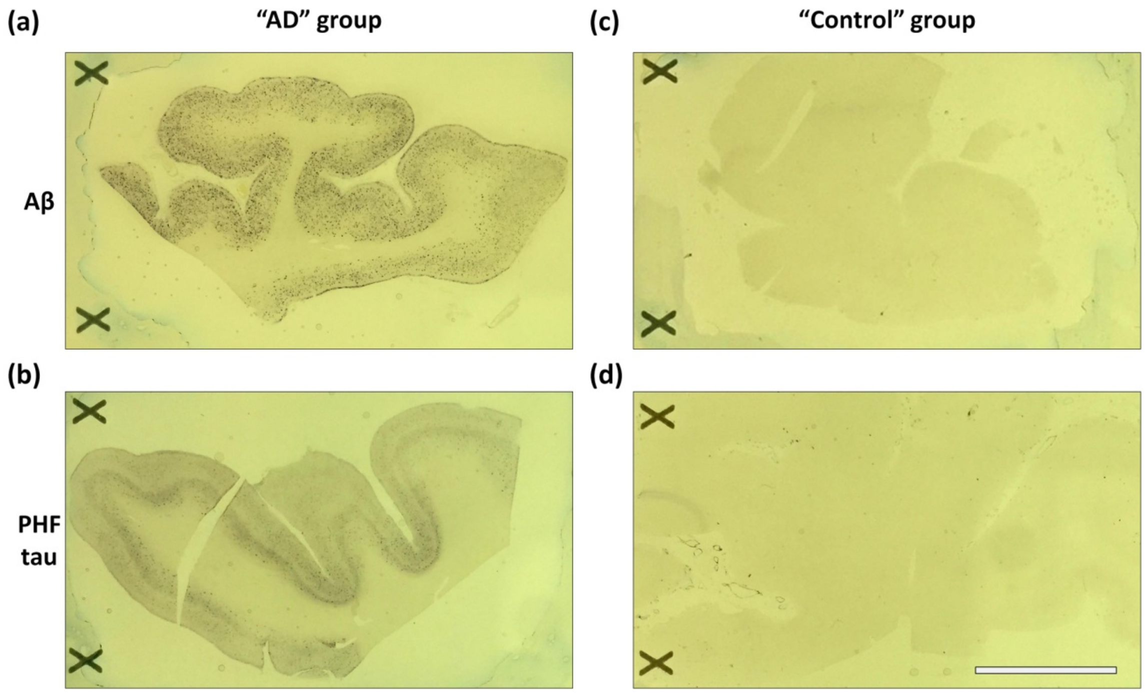

Supplementary Figure 3. Macroscopic evaluation of Aβ and PHF-tau staining in FFPE brain tissue sections. Representative FFPE brain tissue sections from "Alzheimer's disease (AD)" (a,b) and healthy "Control" (c,d) groups were labeled for either Aβ (a,c) or PHF-tau (b,d) pathologic proteins with corresponding 1'Ab/PrA-ssDNA probes and complementary ssDNA'-AP reporters. Localization of labeled proteins was highlighted by a precipitating chromogenic AP substrate (dark staining), and staining patterns were examined macroscopically. Whole-section images were acquired with a hand-held camera. Scale bar, 1 cm.
